# Bioinformatics-Based Identification of a circRNA-miRNA-mRNA Axis in Esophageal Squamous Cell Carcinomas

**DOI:** 10.1155/2020/8813800

**Published:** 2020-09-29

**Authors:** Zhaojun Wang, Haifeng Li, Fajun Li, Xin Su, Junhang Zhang

**Affiliations:** ^1^Department of Thoracic Surgery, The Seventh Affiliated Hospital, Sun Yat-sen University, Shenzhen, China; ^2^Department of Anesthesiology, Guangdong General Hospital, Guangzhou, China; ^3^Department of Critical Care Medicine, The First People's Hospital of Kunshan, Kunshan, China; ^4^Department of Respiratory, Hainan Hospital of PLA General Hospital, Sanya, China

## Abstract

**Background:**

Esophageal squamous cell carcinoma (ESCC) has a poor prognosis due to the lack of early disease symptoms. Using bioinformatics tools, this study aimed to discover differentially expressed nonprotein-coding RNAs and genes with potential prognostic relevance in ESCC.

**Methods:**

Two microRNAs (miRNAs) and one circular RNA (circRNA) microarray datasets were downloaded from the Gene Expression Omnibus (GEO) database. Differential expression of miRNAs (DEMs) and circRNAs (DECs) was, respectively, identified in ESCC tissue and compared to adjacent healthy tissue. Further analysis was performed using the miRNA microarray datasets, where miRTarBase was used to predict which messenger RNAs (mRNAs) was present. This was followed by protein-protein interaction (PPI) network, Kyoto Encyclopedia of Genes and Genomes (KEGG), and Gene Ontology (GO) analyses. Moreover, cytoHubba and UALCAN were used to predict the important nodes and perform patient survival analysis, respectively. The miRNA-associated circRNAs were predicted using the ENCORI website. The interaction between DECs and the predicted circRNAs was also determined. A circRNA-miRNA-mRNA axis was constructed.

**Results:**

Associated with *RAP1B* and circ_0052867, two miRNAs (miR-133b and miR-139-5p) were identified as being differentially expressed and downregulated across the two datasets. Finally, the circ_0052867/miR-139-5p/*RAP1B* regulatory axis was established.

**Conclusion:**

This study provides support for the possible mechanisms of disease progression in ESCC.

## 1. Introduction

Globally, esophageal carcinoma is the eighth most common type of cancer, and the sixth most dominant cause of cancer death [1, 2]. Esophageal squamous cell carcinoma (ESCC) is the major pathology (60–70%) of esophageal cancer (ESCA) [3]. Despite great advances in surgical and radiation therapy, the five-year survival rate of ESCC patients is still under 25%. This is because obvious clinical symptoms of early-stage ESCA are lacking, and many patients are therefore already in an advanced disease stage at the time of diagnosis [3]. A better prognosis is related to the early diagnosis of disease [4]. While molecular markers have been regarded as prognostic markers of ESCC in recent years [5, 6], these biomarkers are not always applicable for early diagnosis or prognosis prediction. Therefore, there is an urgent need to identify new biomarkers to improve the diagnosis and prognosis of ESCC. Biomarkers are stably expressed in plasma, serum, and other body fluids and include circRNAs, miRNAs, and genes [7].

The occurrence and progression of ESCC are related to genetic factors such as genomic amplifications, mutations, insertions, and deletions, as well as tumor epigenetics. The latter includes noncoding RNA, DNA methylation, and histone acetylation [7, 8]. MiRNAs are endogenous, single-stranded, noncoding RNA polymers formed from 20–22 nucleotides and encoded by a single nuclear DNA. They are mainly involved in transcriptional and posttranscriptional regulation of gene expression and bind to complementary sequences of target mRNA. Decreased expression of tumor suppressor miRNA-associated genes and overexpression of oncogenic miRNAs may lead to tumor formation [9]. Recently, miRNAs have been accepted as good prognostic or diagnostic biomarkers in cancer research [10]. Several studies have assessed the possible role of miRNA in identifying the potential for hepatocellular carcinoma metastasis and tumor recurrence [11], and it has also been shown that miRNAs can act on different pathways associated with chemotherapeutic drug sensitivity or resistance in several tumors [12, 13]. Sun et al. found that the level of serum miRNA-1290 could be a useful diagnostic and prognostic marker in ESCC patients [14]. Similarly, miR-455-3p was found to play an antioncogenic role by downregulating *FAM83F* and could be an independent clinical biomarker for predicting the outcome of ESCC patients [15]. One study indicated that decreased miR-145 expression levels could be a useful prognostic marker and could be used to predict overall survival in ESCC patients [16]. The ciRS-7 molecule was shown to be a novel prognostic biomarker and therapeutic target for ESCC [17].

This study aimed to identify differentially expressed miRNAs (DEMs) using GEO datasets, predict which mRNAs were associated to the DEMs, and perform functional enrichment and protein-protein interaction (PPI) network analysis to detect potential disease-associated target genes. The key ESCC-associated genes were identified by survival analysis. The intersection between predicted miRNAs, circRNAs, and differentially expressed circRNAs (DECs) was determined against GEO data, and a potential circRNA-miRNA-mRNA network for ESCC was subsequently established.

## 2. Materials and Methods

### 2.1. Source of the Microarray Data

The public GEO database (http://www.ncbi.nlm.nih.gov/geo) is a functional genomic database from which three ESCC microarray expression datasets (GSE97051, GSE59973, and GSE131969) were downloaded. The circRNA dataset (GSE131969) included data from three ESCC tissue and three adjacent noncancerous tissue samples. The samples were tested against GPL19978 Agilent-069978 Arraystar Human CircRNA microarray V1 data. The miRNA dataset (GSE97051) included data from seven ESCC tissue and seven nonpathogenic esophageal tissue samples that were tested against GPL21572 Agilent-067406 Human CBC lncRNA + mRNA microarray V4.0 data. The miRNA dataset (GSE59973) included three ESCC tissue and three nonpathogenic esophageal tissue samples that were tested against data from the GPL16770 Agilent-031181 Unrestricted_Human_miRNA_V16.0_microarray system.

### 2.2. Identification of DECs and DEMs

Differential analysis was conducted for GSE97051, GSE59973, and GSE131969 to obtain DEMs and DECs between ESCC and healthy control tissue samples by using GEO2R (http://www.ncbi.nlm.nih.gov/geo/geo2r/) [18]. The interaction across miRNA microarray datasets was performed through Venny (http://bioinformatics.psb.ugent.be/webtools/Venn/). Using ENCORI (http://starbase.sysu.edu.cn/index.php), which is an open-source platform for studying RNA interactomes [19], the expression and overall survival of DEMs was examined. The criterion for DECs was an adjusted *p* value <0.05 and |logFC|≧2, while the criterion for DEMs was an adjusted *p* value <0.05 and |logFC|≧1. Volcano plots of the DEMs were created using Sangerbox (http://sangerbox.com/AllTools?tool_id=9699135). The miRTarBase was used to predict potentially significant mRNAs (http://mirtarbase.cuhk.edu.cn/php/index.php).

### 2.3. Functional Enrichment Analysis of Differentially Expressed Genes

GO and KEGG were used to identify the gene functions of individual genomic products and associated pathway information. GO evaluated the cellular component (CC), biological process (BP), and molecular function (MF) of each element [20, 21]. Annotation, visualization, and integrated discovery were performed using the online DAVID database tool (https://david.ncifcrf.gov/). A *p* value <0.05 was considered to be significant.

### 2.4. Construction of Protein-Protein Interaction (PPI) Networks and Validation of the Hub Genes

The STRING database was applied for PPI network construction. Using Cytoscape via the CytoHubba app, the top 50 candidates were considered to be hub genes. To determine the expression of the 50 hub genes in ESCC tissue and to understand the effect(s) of hub genes on prognosis, the expression and survival analyses of hub genes were performed using UALCAN (http://ualcan.path.uab.edu/index.html). The miRNA-mRNA subnetwork of interest was determined. *p* < 0.05 was considered to indicate statistical significance.

### 2.5. Validation of the circRNAs and Construction of the Competitive Endogenous RNA Network

StarBase 3.0 was introduced to predict linkage between the miRNAs and circRNAs. Using Venny against the GEO dataset, DECs of interest were predicted. The intersection between circRNAs and these DECs indicated the target circRNAs in ESCC tissue. Finally, a circRNA-miRNA-mRNA network was constructed, and the data were visualized using Cytoscape software 3.7.1.

## 3. Results

### 3.1. Two Potential miRNAs Selected for ESCC

**Figure 1:**
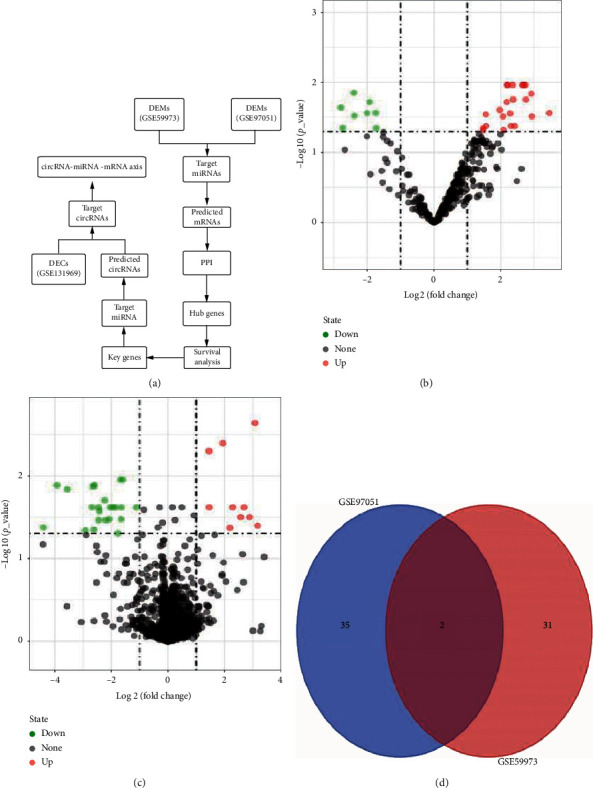
Flow chart of the approach and identification of potential miRNAs in ESCA (esophageal cancer). (a) Flow chart of the approach utilized in this study. DEMs: differential expression of miRNAs and DECs: differential expression of circRNAs. (b) The volcano plot of differentially expressed miRNAs from GSE59973 dataset. (c) The volcano plot of differentially expressed miRNAs from GSE97051 dataset. (d) The intersection analysis of DEMs from GSE59973 dataset and GSE97051 dataset.

**Figure 2:**
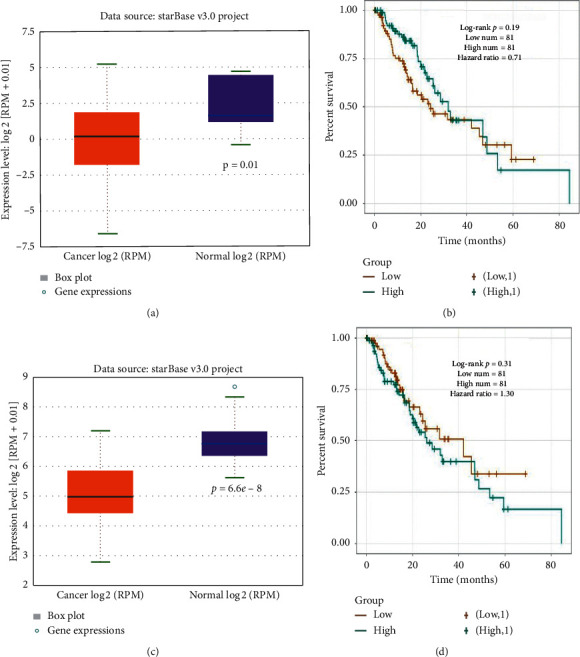
The expression level and overall survival of miRNAs in ESCA (esophageal cancer). (a) The expression level of hsa-miR-133b with 162 cancer and 11 normal samples in ESCA. (b) Overall survival for hsa-miR-133b in ESCA cancer. (c) The expression level of hsa-miR-139-5p with 162 cancer and 11 normal samples in ESCA. (d) Overall survival for hsa-miR-139-5p in ESCA cancer. *p* < 0.05 represents significant difference.

To find the potential miRNA biomarkers in ESCC, two miRNA datasets (GSE97051 and GSE59973) were selected (Table 1). As per the selection criteria, and as shown in Figure 1(d), two downregulated DEMs (miR-133b and miR-139-5p) were isolated. Next, the expression and overall survival of two downregulated DEMs in ESCA data were examined using ENCORI. Both miR-133b (*p*=0.01) and miR-139-5p (*p*=6.6*e* − 8) showed significantly low expression in cancer samples, but no effect on overall patient survival was seen in ESCA (Figure 2).

### 3.2. Prediction and Analysis of mRNAs Binding to the Potential ESCC-Associated miRNAs

**Figure 3:**
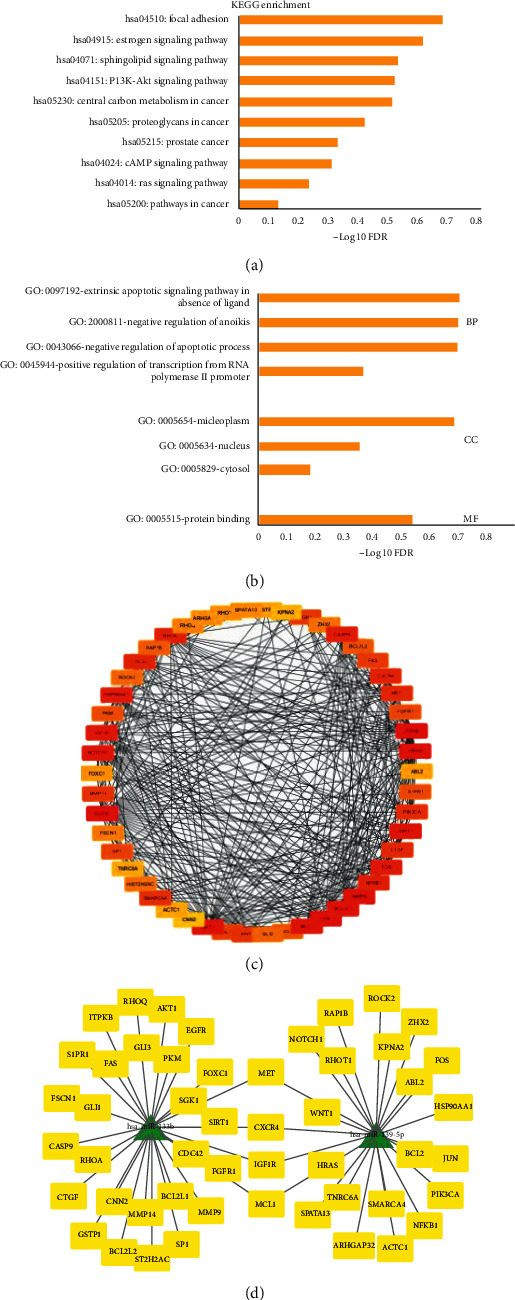
Enrichment analysis of target genes, identification of hub genes, and the network of miRNA-target genes. (a) KEGG analyses. (b) GO analyses. (c) The top 50 hub genes were selected CytoHubba. (d) The network of miRNA-target genes.

The miRTarBase database was used to identify which mRNAs might bind to the two predicted miRNAs. In total, 87 mRNAs potentially bind to miR-133b, and 105 mRNAs potentially bind to miR-139-5p. In order to identify key nodes in the PPI network, the top 50 hub genes were selected using CytoHubba based on the STRING database (Figure 3(c) and Table S1). For better visualization, the miRNA-mRNA connecting network was constructed using Cytoscape (Figure 3(d)).

### 3.3. Enrichment Analysis of the Target Genes

KEGG enrichment and GO analyses were performed for the target genes of the two DEMs (Figure 2). In the BP category, the “extrinsic apoptotic signaling pathway in absence of ligand,” “negative regulation of anoikis,” and “negative regulation of apoptotic process” were enriched. While “protein binding” was enriched according to the MF category, enrichment of the “nucleoplasm,” “nucleus,” and “cytosol” was shown in the CC category (Figure 3(b)). The KEGG pathway analysis revealed enrichment of the “focal adhesion,” “estrogen signaling,” “sphingolipid signaling,” and “PI3K-Akt signaling” pathways (Figure 3(a)).

### 3.4. Identification of mRNA Influences Patient Survival

To analyze the 50 mRNAs selected by the PPI network, survival analysis was performed using UALCAN. It was found that only *RAP1B* expression was significantly increased in ESCA tumor samples (*p*=2.3529*e* − 4). Additionally, the higher the expression of *RAP1B*, the worse the patient's survival (*p*=0.011). The miR-139-5p/*RAP1B* subnetwork was therefore determined.

### 3.5. Validation of the circRNAs and Construction of the Competitive Endogenous RNA Network

**Figure 4:**
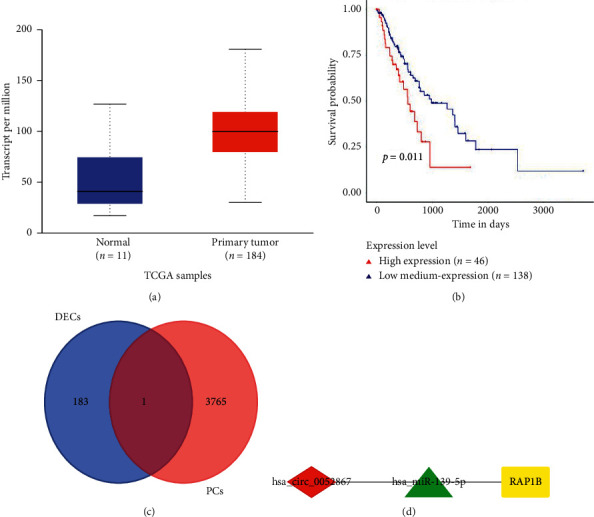
The network of circRNA-miRNA-target genes. (a) The expression level of *RAP1B* in ESCA based on sample type. (b) Effect of *RAP1B* expression level on ESCA patient survival. (c) The intersection analysis of DECs and PCs. DECs: differential expression of circRNAs and PCs: predicted circRNAs. (d) The network of circRNA-miRNA-target genes axis. *p* < 0.05 represents significant difference.

ENCORI was utilized to identify the 3766 potential circRNAs targeted by miR-139-5p. The intersection of these circRNAs and the predicted DECs found circ_0052867 to be of interest (Figure 4(c)). Finally, the circ_0052867/miR-139-5p/*RAP1B* network was constructed (Figure 4(d)) and was concluded to be a circRNA-miRNA-mRNA regulatory axis.

## 4. Discussion

Due to the absence of obvious clinical symptoms in the early stages of ESCC and the lack of sensitive early diagnostic detection methods, most patients with ESCC are diagnosed at an advanced disease stage. Consequently, the best treatment opportunity is lost. Stein et al. reported that the five-year survival rate for early ESCA was 62.9% following surgery [22]. Nevertheless, the validity and sensitivity in detecting traditional tumor markers are insufficient for early ESCC diagnoses [23]. There is therefore an urgent need for improved biomarkers for early detection of ESCC.

Playing an important role in pathological and physiological processes, noncoding RNA comprises approximately 98% of the human genome [24]. Many studies have demonstrated that competitive endogenous (ceRNAs) are able to act as sponges for miRNAs [25], while miRNAs have been shown to play an important role in tumorigenesis and its prognosis [26]. Li et al. found that the survival time of high miRNA-506 expression was significantly shorter than that of low miRNA-506 expression [27]. Downregulated miRNA-718 expression may also serve as a potential diagnostic marker for ESCC [28].

The circRNAs are newly found noncoding RNAs that participate in cancer development [29] and are currently being investigated as potential cancer biomarkers [30, 31]. Leading to poor prognosis in ESCC, upregulated circRNA_100876 expression can accelerate cell proliferation and metastasis [32], while the upregulation of circRNA_100873 is associated with increased lymphatic metastasis in ESCC [33]. A further study reported that circGSK3 overexpression enhances multiple metastasis and invasion of the tumor [34]. The underlying mechanisms of circRNAs and miRNAs in ESCC patient survival remain unknown.

Real-time quantitative PCR (q-PCR) is a classical method to detect abnormal noncoding RNA expression in ESCC tissue. Next generation sequencing techniques and available microarray data provide researchers with a more complete noncoding RNA expression profile between ESCC tumor and nonpathogenic tissue samples. For example, Yang et al. detected 39 dysregulated miRNAs (28 downregulated and 11 upregulated) in tumor/nonpathogenic ESCC tissue [35]. Serum miRNA expression profiles were detected in 52 ESCC patients, and three miRNAs were identified as biomarkers for the diagnosis of ESCC [36].

In the present study, the intersection between two GEO databases confirmed miR-133b and miR-139-5p to be of interest, with an miRNA-mRNA-circRNA interaction being predicted. According to ceRNA theory, the expressions of miRNAs should be negatively correlated with the expressions of its targeted mRNAs and circRNAs. In this study, two downregulated DEMs were predicted to be associated with upregulated mRNA and circRNA targets. The PPI network was used to identify hub genes, and survival analysis was performed in order to find the key genes. Subsequently, *RAP1B* and circ_0052867 were found to have an impact on the survival of ESCC patients. Consequently, we constructed the circ_0052867/miR-139-5p/*RAP1B* ceRNA regulatory network, which may be a potential biomarker for ESCC patient survival. To our knowledge, this is the first study to report the expression of circ_0052867 in ESCC. While it has been found that the expression level of miR-139-5P in 11 cases of esophageal cancer is significantly higher than that of adjacent paracarcinoma tissue [37]. Yang et al. reported that hsa-miR-139-5p was downregulated in ESCC tumor tissue compared to nontumor tissue samples [38]. Jia et al. found that the expression of *RAP1B* was upregulated in ESCC tissue, and *RAP1B* promoted the growth, migration, and metastasis of the ESCC cells [39]. However, Zhang et al. concluded that miR-518b may play an anticancer role in the development of ESCC by targeting *RAP1B* [40]. Nevertheless, the exact mechanism remains to be discovered.

There were still some limitations to this study. Firstly, the findings of this study require further experimental verification. Secondly, the sample size is too small, and there are only a limited number of samples in each microarray dataset. Lastly, as mRNAs that influence patient survival and miRNAs that alter expression in ESCC tissue were identified in a database which includes ESCA tumor samples, there may be bias towards ESCC patients in this analysis.

## Figures and Tables

**Table 1:** Basic information of the three microarray datasets from GEO.

Data source	Platform	Series	Sample size (T/N)
circRNA	GPL19978	GSE131969	3/3
miRNA	GPL16770	GSE59973	3/3
miRNA	GPL21572	GSE97051	7/7

*Note*. T: tumor; N: normal.

## Data Availability

The data used to support this study are available in TCGA database and GEO database.

## Supplementary Materials

Supplementary Materials Table S1: 50 hub genes selected using CytoHubba ranked by MCC method.
