# Supplementary material for: Bioinformatics-Based Identification of a circRNA-miRNA-mRNA Axis in Esophageal Squamous Cell Carcinomas
Source: J Oncol. 2020 Sep 29;2020:8813800. doi: 10.1155/2020/8813800 (PMC7542503; doi:10.1155/2020/8813800)
Supplement: Supplementary Materials — Table S1: 50 hub genes selected using CytoHubba ranked by MCC method. [file 8813800.f1.docx]

Table S1. 50 hub genes selected using CytoHubba ranked by MCC method.

| Name | Score |
| --- | --- |
| EGFR | 3.85E+08 |
| ITPKB | 3.85E+08 |
| JUN | 3.82E+08 |
| MMP9 | 3.82E+08 |
| HRAS | 3.81E+08 |
| AKT1 | 3.73E+08 |
| BCL2L1 | 3.28E+08 |
| MCL1 | 3.24E+08 |
| SIRT1 | 3.21E+08 |
| NOTCH1 | 2.25E+08 |
| IGF1R | 2.20E+08 |
| FOS | 2.08E+08 |
| HSP90AA1 | 1.73E+08 |
| CASP9 | 1.60E+08 |
| NFKB1 | 1.60E+08 |
| RHOA | 5.60E+07 |
| CDC42 | 5.60E+07 |
| CXCR4 | 5.23E+07 |
| MET | 1.20E+07 |
| CTGF | 4400907 |
| PIK3CA | 2958294 |
| SMARCA4 | 47071 |
| WNT1 | 46323 |
| MMP14 | 45364 |
| SGK1 | 11761 |
| FAS | 11640 |
| GLI1 | 6160 |
| SP1 | 2399 |
| FGFR1 | 2336 |
| S1PR1 | 1441 |
| PKM | 869 |
| BCL2 | 734 |
| BCL2L2 | 726 |
| RAP1B | 268 |
| GLI3 | 248 |
| ROCK2 | 168 |
| HIST2H2AC | 139 |
| ZHX2 | 120 |
| FSCN1 | 43 |
| RHOQ | 36 |
| SPATA13 | 30 |
| ARHGAP32 | 30 |
| FOXC1 | 25 |
| RHOT1 | 25 |
| ABL2 | 24 |
| GSTP1 | 24 |
| ACTC1 | 22 |
| TNRC6A | 22 |
| KPNA2 | 17 |
| CNN2 | 14 |
